# An evidential reasoning based model for diagnosis of lymph node metastasis in gastric cancer

**DOI:** 10.1186/1472-6947-13-123

**Published:** 2013-11-06

**Authors:** Zhi-Guo Zhou, Fang Liu, Li-Cheng Jiao, Zhi-Long Wang, Xiao-Peng Zhang, Xiao-Dong Wang, Xiao-Zhuo Luo

**Affiliations:** 1 School of Computer Science and Technology, Xidian University, Xi’an 710071, P. R. China; 2 Key Laboratory of Intelligent Perception and Image Understanding of Ministry of Education of China, Xidian University, Xi’an 710071, P. R. China; 3 Key Laboratory of Carcinogenesis and Translational Research of Ministry of Education of China, Department of Radiology, Peking University Cancer Hospital & Institute, Beijing 100142, P. R. China

**Keywords:** Gastric cancer, Lymph node metastasis, Evidential reasoning

## Abstract

**Background:**

Lymph node metastasis (LNM) in gastric cancer is a very important prognostic factor affecting long-term survival. Currently, several common imaging techniques are used to evaluate the lymph node status. However, they are incapable of achieving both high sensitivity and specificity simultaneously. In order to deal with this complex issue, a new evidential reasoning (ER) based model is proposed to support diagnosis of LNM in gastric cancer.

**Methods:**

There are 175 consecutive patients who went through multidetector computed tomography (MDCT) consecutively before the surgery. Eight indicators, which are serosal invasion, tumor classification, tumor enhancement pattern, tumor thickness, number of lymph nodes, maximum lymph node size, lymph node station and lymph node enhancement are utilized to evaluate the tumor and lymph node through CT images. All of the above indicators reflect the biological behavior of gastric cancer. An ER based model is constructed by taking the above indicators as input index. The output index determines whether LNM occurs for the patients, which is decided by the surgery and histopathology. A technique called k-fold cross-validation is used for training and testing the new model. The diagnostic capability of LNM is evaluated by receiver operating characteristic (ROC) curves. A Radiologist classifies LNM by adopting lymph node size for comparison.

**Results:**

134 out of 175 cases are cases of LNM, and the remains are not. Eight indicators have statistically significant difference between the positive and negative groups. The sensitivity, specificity and AUC of the ER based model are 88.41%, 77.57% and 0.813, respectively. However, for the radiologist evaluating LNM by maximum lymph node size, the corresponding values are only 63.4%, 75.6% and 0.757. Therefore, the proposed model can obtain better performance than the radiologist. Besides, the proposed model also outperforms other machine learning methods.

**Conclusions:**

According to the biological behavior information of gastric cancer, the ER based model can diagnose LNM effectively and preoperatively.

## Background

Gastric cancer has become one of the major causes of cancer-related deaths in the world [1]. Lymph node metastasis (LNM) is a very important prognostic factor regarding long-term survival [2]. The TNM^a^ staging system based on American Joint Committee on Cancer is taken as the evaluated standard and has been widely accepted [3]. Based on this standard, the 5-year survival rate of patients in N0 stage after surgery is 86.1%, while N1, N2 and N3 stage patients can obtain 58.1%, 23.3% and 5.9%, respectively [4].

Currently, doctors diagnose LNM empirically based on the size of lymph nodes which relies on various imaging methods, such as endoscopic ultrasound (EUS), abdominal ultrasound, multi-slice spiral computerized tomography (CT), Magnetic Resonance Imaging (MRI) and Positron Emission computed Tomography (PET). However, none of the above imaging tools can acquire the lymph node status in a satisfactory way. Meanwhile, a systemic review shows that EUS, MDCT, conventional MRI, and FDG-PET cannot be used to confirm or exclude the presence of LNM reliably [2]. The reason is that large lymph nodes may be caused by inflammation, while small ones may be caused by metastasis. Therefore, single lymph node size is not a strong predictor. In fact, many studies have shown that LNM is related to tumor size, pathological lymphatic involvement, histological type and other factors [5-8]. Therefore, a method which combines lymph node size with these factors should be considered. Furthermore, a few researches [9-11] have discussed the diagnostic capabilities of morphological characteristics in rectum cancer. According to these studies, the morphological characteristics including border contour and signal intensity of lymph nodes may partly improve the diagnostic ability of metastasis. However, these studies mainly focus on the MRI imaging in rectum cancer. For patients with gastric cancer in clinical practice, abdomen CT is a more common used imaging modality than MRI examination. Hence, we consider building a model to diagnose LNM with multiple indicators.

As there are qualitative and quantitative data in eight indicators, a method which can integrate these two types of data should be adopted. The ER approach was originally proposed to deal with multiple attribute decision analysis problems that involve both qualitative and quantitative attributes under uncertainty [12]. The kernel is the ER algorithm which is developed on the basis of the decision theory and the Dempster-Shafer (D-S) theory of evidence [13,14]. As ER can integrate the qualitative information and quantitative data reasonably, it is applied. One of the aims in this paper is to analyze which indicators are related to the biological behavior of gastric cancer and construct a mathematical model to assess LNM preoperatively.

## Methods

### Patients

In this experiment, 175 CT cases obtained from Peking University Cancer Hospital & Institute (Beijing, China P. R.) constitute the sample set. According to the international treatment guideline of gastric cancer, CT is one of the most commonly used inspections [15]. However, other methods such as PET and EUS are used as selected check. These patients were administered preoperative contrast enhancement abdominal in the CT examinations and received the gastrectomy between April 2006 and September 2008. This retrospective study was approved by institutional review board (IRB). They were preoperatively examined with MDCT. Note that we have obtained the informed consent from all selected patients prior to the routine clinical course of CT examinations. There are 125 males and 50 females among these patients, and their average age is 59.8 years. The details are shown in Table 1.

**Table 1:** Patient characteristics

**Clinic pathological features**	**Value**
Number of patients	175
Average age(y)	59.8(30-85)
Ratio of men to women	125:50
Histopathology	
Adenocarcinoma	173(98.9%)
Well differentiated	6(3.4%)
Moderately differentiated	91(52%)
Poorly differentiated	76(43.5%)
Small cell carcinoma	2(1.1%)
lymph node metastasis	
Positive	134(76.6%)
Negative	41(23.4%)

### Indicators

There are eight indicators which were extracted by two radiologists, one with three years and another with eight years experience in abdominal CT. The eight indicators were measured and counted manually on MDCT images as follows:

(1) Serosal invasion: Axial and MPR images are evaluated to determine the serosal invasion simultaneously. The entire thickening stomach wall abnormally enhances linear or reticular structures in the fatty layer surrounding the stomach indicated serosal invasion [16].

(2) Tumor classification: Early gastric cancer or Bormann classification of advanced cancer in MPR images is confirmed.

(3) Tumor enhancement pattern: Tumor enhancement is divided into three patterns at portal phase of CT images, which are mucosal surface enhancement, homogeneous enhancement and heterogeneous enhancement.

(4) Tumor thickness: The maximal thickness of tumor is measured at the axial CT images.

(5) The number of lymph nodes: The number of the gastric regional lymph nodes with size larger than 3 mm in MDCT images by groups is counted [17]. As the lymph nodes, which are smaller than 3 mm, are too tiny to make them discernible, they are omitted.

(6) Maximum lymph node size: The short axis of the largest lymph node detected in CT images is measured.

(7) Lymph node station: The lymph node station with MDCT images based on the Japanese classification of gastric carcinoma is determined [17].

(8) Lymph node enhancement: It means CT attenuation value of lymph node, which is measured at the portal venous phase of CT image.

In this paper, all the indicators are measured manually. The number of lymph nodes is the amount of lymph nodes around the stomach. Maximum lymph node size and lymph node enhancement is extracted from the maximal lymph node. The objective is to predict whether LNM occurs other than maximum lymph node has LNM. In other words, the object is to predict whether LNM occurs for each patient. The final result for LNM diagnosis is decided by the surgery and histopathology. The pathological result can definitely confirm whether LNM occurs or not. We do not want to predict metastasis for each lymph node. The reason is that one-to-one lymph node’s correspondence with CT and pathology depends on very precise and excellent experience of radiologist. It is usually not consistent adequately for different radiologists, which may affect the prediction accuracy of the mathematical model. Therefore, we did not make the one-to-one correspondence for every lymph node. The details are described in Table 2.

**Table 2:** Description of eight indicators

**Patient data**	**LNM(-)**	**LNM(+)**
Patient number	41/175	134/175
Measurement data		
Tumor thickness(mm)	13.3 ± 14.0	16.6 ± 28.4
Maximum lymph node size(mm)	6.5 ± 2.8	10.0 ± 5.5
The number of lymph nodes	7 ± 4	12 ± 8
Lymph node enhancement	39.5 ± 58.5	62.5 ± 66.5
Count data		
Tumor enhancement pattern		
Pattern 1	13/175	6/175
Pattern 2	26/175	118/175
Pattern 3	2/175	10/175
Serosal invasion		
Yes	15/175	120/175
No	26/175	14/175
Tumor classification		
Early gastric cancer	9/175	1/175
Borrmann I	2/175	0/175
Borrmann II	3/175	9/175
Borrmann III	27/175	121/175
Borrmann IV	0/175	3/175
Lymph nodes station		
Station 1	29/175	44/175
Station 2	12/175	54/175
Station 3	0/175	36/175

The value of the measurement data was measured manually, and the count data was the number of data.

### ER based model

In this model, we represent every case by an over-complete dictionary whose elements are the training samples. If sufficient training samples are available from each class, it will be possible to represent the test sample.

Assume that training samples are denoted by *X = {X*_*1*_*,X*_*2*_*,…,X*_*p*_*}*∈ *R*^*mxn*^, where *n* is the number of training samples, and *m* is the number of indicators. *y*∈*{1,2,…,p}* is the label and *p* is the class index. *T = [T*_*1*_*,T*_*2*_*…,T*_*m*_*]*^*T*^ denotes a test sample. The over-complete dictionary *A* is denoted as follows:

(1) $$A=\left[\begin{array}{l} A_{1,1},A_{1,2},\ldots ,A_{1,n}\\ A_{2,1},A_{2,2},\ldots ,A_{2,n}\\ \ldots \;,\;\ldots \;,\ldots ,\;\ldots \;\\ A_{m,1},A_{m,2},\ldots ,A_{m,n} \end{array}\right]$$

Here *A* consists of training samples and *A*_*m,n*_ represents every indicator in training samples. According to the limits of ER, the columns of *A* and *T* should be normalized firstly. Then each indicator T_*i*_ in test sample is represented by *A* and corresponding coefficients *w*_*i*_*,i* = 1,2,…,*m*. Then we utilize the ER analytic algorithm [13] as follows:

(2) $$T_{j}=\frac{\mu \times \left[\prod_{k=1}^{n}\left(\omega_{k}A_{j,k}+1-\omega_{k}\sum_{i=1}^{m}A_{i,k}\right)-\prod_{k=1}^{n}\left(1-\omega_{k}\sum_{i=1}^{m}A_{i,k}\right)\right]}{1-\mu \times \left[\prod_{k=1}^{n}\left(1-\omega_{k}\right)\right]}$$

(3) $$\begin{array}{l} \mu =[\sum_{j=1}^{m}\prod_{k=1}^{n}\left(\omega_{k}A_{j,k}+1-\omega_{k}\sum_{i=1}^{m}A_{i,k}\right)\\ \;{-\left(m-1\right)\overset{n}{\underset{k=1}{\prod }}\left(1‒\omega_{k}\sum_{i=1}^{m}A_{i,k}\right)]}^{-1} \end{array}$$

All the indicators in *T* can be represented by *A* and *w*_*i*_,*i* = 1,2,…,*m* using the ER approach. Assume that *ER* represents the ER approach. Therefore, *T* is represented as follows:

(4) $$T=\mathrm{ER}\left(A,\omega \right)$$

where ω∈*R*^*n*^ is the coefficient vector. However, it is not possible to guarantee the optimal solution and instead we replace it by the approximate solution provided in Equation (5):

(5) $$T\approx \mathrm{ER}\left(A,\omega \right)$$

As the new case can be sufficiently represented by the training samples from the same class, we obtain the prediction by ω. The objective is to minimize residual between expectation output and observed output. We solve this problem by calculating the following *l*_*2*_ minimization problem:

(6) $$\underset{\omega }{\text{min}}||T-\mathrm{ER}\left(A,\omega \right)||{}_{2}^{2}$$

where ω is the parameter that needs to be optimized. The constrained condition is,

(7) $$\sum_{i=1}^{n}\omega_{i}=1,\omega_{i}\geq 0,i=1,\ldots ,n$$

The commonly used single-objective optimization methods with constraint handling can be utilized. In this paper, *FMINCON* function in MATLAB toolbox is adopted.

Then the residual *r*_*j*_,*j* = 1,…,*n* is calculated between *y* and each class in *T* as Equation (8). The residual is calculated in the *l*_*2*_*-*norm. For each class *j*, let *w*_*j*_ be the characteristic function that selects the coefficients associated with the *j*th class. When using the coefficients associated with the *j*th class, one can approximate the given test sample y as *ŷ*_*j*_ = *ER*(*T,w*_*j*_).

(8) $$r_{j}\left(T\right)=||T-\mathrm{ER}\left(\omega ,A_{j}\right)||{}_{2}^{2},j=1,\ldots ,p$$

Finally, we can classify *T* by assigning it to the object class that minimizes the residual as follows:

(9) $$\mathrm{identity}\left(T\right)=\arg\text{min}r_{j}\left(T\right),j=1,\ldots ,p$$

The prediction result is the same as the class with minimum residual. We summarize the procedures as follows:

Step 1: Input dictionary *A* = [*A*_1_,*A*_2_,…,*A*_*k*_]∈*R*^*mxn*^ and a new case *T*∈*R*^m^.

Step 2: Normalize the columns of *A* and *y* to have unit *l*_*2*_-norm.

Step 3: Solve the *l*_*2*_-minimization problem to obtain ωas in Equation (6).

Step 4: Calculate the residuals *r*_*j*_*(T)* as Equation (8).

Step 5: Output the final label for which *r*_*j*_*(T)* is minimized.

## Results

By univariate statistical analysis, it shows that all the indicators including serosal invasion, tumor classification, tumor enhancement pattern, tumor thickness, number of lymph nodes, maximum lymph node size, lymph nodes station and Lymph node enhancement are significant different between LNM positive and negative group (P < 0.001). On the other hand, 5-fold cross validation is used in all the experiments [18].

**Figure 1:**
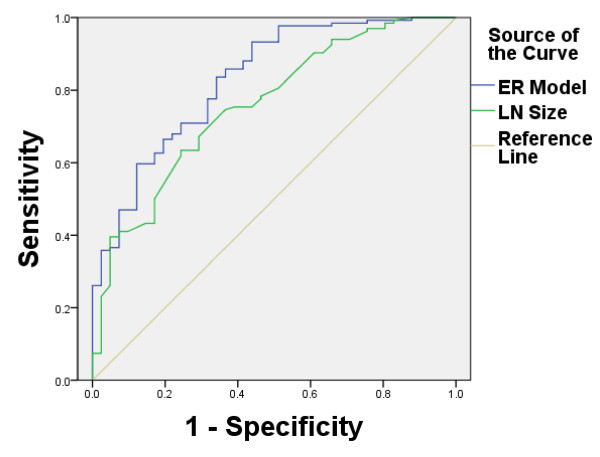
**ROC curve between ER based model and radiologist.** Receiver operating characteristic (ROC) curve for LNM with ER based model and the radiologist. The AUC of the proposed model is 0.829, while the radiologist is 0.757.

Taking 7.7 mm as the best cut-off point of maximum lymph node size, the radiologist achieves an AUC of 0.757 for evaluating LNM. In other words, if lymph node size is larger than 7.7 mm, it is considered that LNM occurs; otherwise, it does not occur. The sensitivity and specificity are only 63.4% and 75.6%. However, the sensitivity, specificity and AUC of the proposed model are 89.51%, 80%, and 0.829, respectively (Figure 1 and Table 3). According to statistical analysis, the ER based model performs significantly better (P < 0.05) than radiologists.

**Table 3:** AUC values of nine methods on training data

**Model**	**Area**	**Std error**	**Asymptotic sig.**	**Asymptotic 95% confidence interval**	
				**Lower bounder**	**Upper bounder**
ER based model	0.948	0.020	0.000	0.908	0.988
ANN	0.798	0.043	0.000	0.713	0.882
SVM linear	0.944	0.019	0.000	0.906	0.981
SVM Gaussian	0.955	0.018	0.000	0.920	0.990
SVM Ploynomial2	0.94	0.022	0.000	0.898	0.983
SVM Ploynomial3	0.938	0.022	0.000	0.894	0.982
SVM Ploynomial4	0.941	0.022	0.000	0.898	0.983
Logistic Regression	0.888	0.027	0.000	0.835	0.940

**Figure 2:**
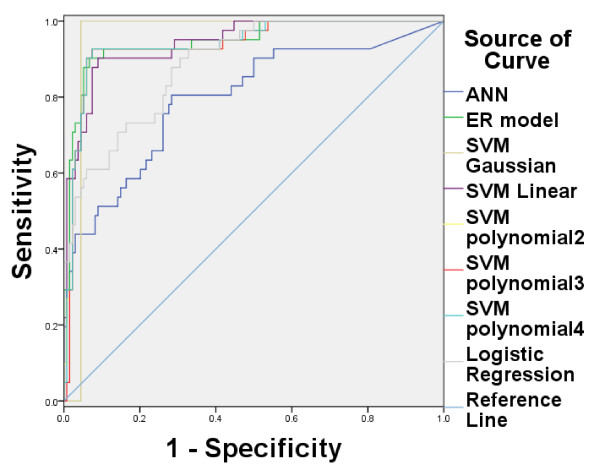
**ROC curve among eight methods on training data.** Figure 2 shows the ROC curve for six methods on training data. The AUC of SVM whose kernel function is Gaussian is the largest among eight methods. The ER based model is a slightly lower than Gaussian kernel. However, it is better than other six methods.

**Figure 3:**
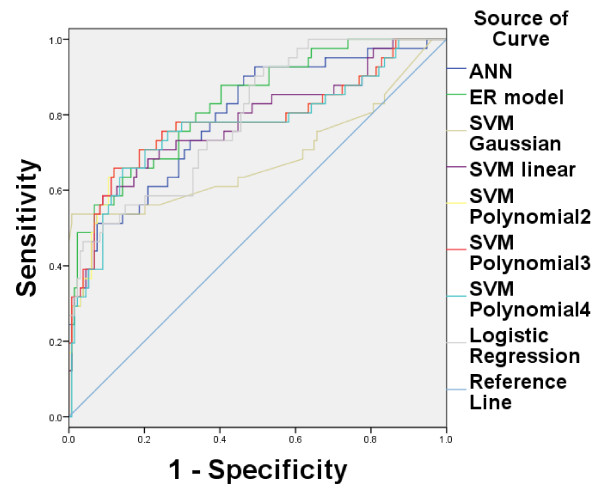
**ROC curve among eight methods on testing data.** Figure 2 shows the ROC curve for six methods on testing data. The AUC of the ER based model is the largest among six methods.

On the other hand, there are many other machine learning methods that can be used for evaluating LNM. Some typical methods are used for comparisons, including Artificial Neural Network (ANN) [19,20], Support Vector Machine (SVM) and logistic regression. For SVM, a linear, a Gaussian and a polynomial kernel are tested, and LibSVM2.91 is used [21]. The linear SVM is named as SVM linear. For Gaussian kernel (named as SVM Gaussian), the regularization and kernel parameters are set as {2^-3^,2^-2^,…,2^10^}, and the highest recognition rate is regarded as the output. For polynomial kernel, three degrees such as 2 (named as SVM polynomial2), 3 (named as SVM polynomial3), and 4 (named as SVM polynomial4) are tested. The feed forward neural network in MATLAB toolbox is adopted for ANN which has a single hidden layer and the number of nodes is 5. Binary logistic regression in SPSS is used for logistic regression. 5-fold-cross validation is still used in all studies. Figure 2 shows the ROC and Table 3 shows the sensitivity, specificity and AUC of these nine methods on training data, while Figure 3 and Table 4 show the results on testing data. The experimental results show that ER based model can obtain better performance than other commonly used machine learning methods.

**Table 4:** AUC values of nine methods on testing data

**Model**	**Sensitivity**	**Specificity**	**AUC**^**1**^	**P value** **(AUC compared with Radiologist)**
ER based model	0.8951	0.8	0.829 ± 0.037	P < 0.001
ANN	0.8453	0.6429	0.791 ± 0.041	P < 0.001
SVM Linear	0.8723	0.6765	0.78 ± 0.046	P < 0.001
SVM Gaussion	0.7657	0.6036	0.688 ± 0.059	P < 0.001
SVM Ploynomial2	0.8611	0.6774	0.781 ± 0.048	P < 0.001
SVM Ploynomial3	0.8662	0.6667	0.782 ± 0.048	P < 0.001
SVM Ploynomial4	0.8633	0.6111	0.769 ± 0.049	P < 0.001
Logistic Regression	0.8552	0.6667	0.793 ± 0.039	P < 0.001
Radiologist^2^	0.634	0.765	0.757 ± 0.042	

^1^The value of data was AUC + standard deviation.

^2^Taking 7.7 mm as the best cut-off point of maximum lymph node size, and if lymph node size larger than 7.7 mm, it is considered that LNM occurs; otherwise, it does not occur.

## Discussion

LNM has a great effect on the surgical treatment of patients with gastric cancer and is an important factor in prognosis. Currently, the standard for judging LNM mainly depends on morphological indicators and lymph node size is the dominant indicator. However, there are some different opinions. For example, Fukuya T et al. [22] showed CT attenuation and lymph node configuration could aid in diagnosis of malignant adenopathy. Deutsch SJ et al. [23] pointed out that location, size, density and contour are not helpful in distinguish benign from malignant lymphadenopathy. Therefore, the main constraint is that there is no unified criterion for evaluating LNM preoperatively.

Several other methods are applied to evaluate LNM. The commonly used method is artificial neural network (ANN). For example, Bollschweiler et al applied a single-layer perception to predict LNM in gastric cancer and the accuracy is 79% [24]. However, it has some deficiencies: 1) ANN is always sensitive to initial parameters and needs to spend more time evaluating them. 2), it is prone to be over fitting. Besides this, other machine learning methods are also used for evaluating LNM. Compared with ANN, the new model is generated without assuming system structure or parameters a prior, thus no parameters needs to be initialized, which can help circumvent the bottleneck of estimating all initial parameters. Moreover, the ER based model can obtain a higher prediction performance among the presented methods. Therefore, it seems that ER based model is a suitable method for evaluating LNM.

## Conclusions

By utilizing the biological behavior information of gastric cancer on CT images, the proposed ER based model can help effectively diagnose the LNM preoperatively.

## Endnote

^a^The international normative TNM classification describes the state of the tumor (T), the lymph nodes (N), and possible metastases (M).

## Competing interests

The authors declare that they have no competing interests.

## Authors’ contributions

ZGZ, FL and LCJ carried out the study design. ZGZ carried out the manuscript editing. ZLW and XPZ carried out the data acquisition and interpretation. XDW and XZL participated in the design of the study and the statistical analysis. All authors read and approved the final manuscript.

## Pre-publication history

The pre-publication history for this paper can be accessed here:

http://www.biomedcentral.com/1472-6947/13/123/prepub
